# Life Course Socioeconomic Status and Later Life Alzheimer’s Disease-Related Neuropathological Lesions

**DOI:** 10.1093/geroni/igaa057.532

**Published:** 2020-12-16

**Authors:** Sarah Tom, Amol Mehta, Stepanie Izard, Paul Crane, David Bennett, Philip De Jager, Julie Schneider

**Affiliations:** 1 Columbia University, New York, New York, United States; 2 University of Washington, Seattle, Washington, United States; 3 Rush University Medical Center, Chicago, Illinois, United States; 4 Rush Alzheimer's Disease Center, Chicago, Illinois, United States

## Abstract

While higher life course socioeconomic status (SES) is associated with lower Alzheimer’s Disease (AD) risk, relationships with AD-related neuropathological lesions are unclear. We hypothesize that high SES in early, mid and late life will be associated with lower frequency of AD-related pathological lesions. The Rush Memory and Aging Project is a cohort of 2025 people age ≥ 65 years from Northeastern Illinois recruited 1997 – 2018; 972 participants died. We created binary variables for Braak stage (0-II versus III-VI), NIA-Reagan score (low likelihood/no AD pathology versus high/intermediate likelihood), presence of microinfarcts and, separately, macroinfarcts, and life course SES based on median for late life (baseline income), midlife (income at age 40 years), and early life (composite of parental education and number of siblings). Logistic regression models adjusted for ages at baseline and death, sex, presence of APOE-Ɛ4 alleles, and separately, vascular factors and education. Of 761 participants with relevant data, 69% were women, and mean ages at baseline and death were 83 + 6 years and 90 + 6 years, respectively. High early life SES was related to lower frequency of AD pathology (OR= 0.69, 95% CI 0.50, 0.96) and macroinfarcts (OR= 0.69, 95% CI 0.51, 0.94). Results were similar when adjusting for vascular factors; adjustment for education modestly attenuated these associations. Mid-life and late life SES were not associated with AD-related neuropathological lesions. High early life SES was related to lower frequency of AD pathology and macroinfarct presence. Environment during early development may influence later life brain aging.

